# The Relationship between Livestock Ownership and Child Stunting in Three Countries in Eastern Africa Using National Survey Data

**DOI:** 10.1371/journal.pone.0136686

**Published:** 2015-09-11

**Authors:** Emily M. Mosites, Peter M. Rabinowitz, Samuel M. Thumbi, Joel M. Montgomery, Guy H. Palmer, Susanne May, Ali Rowhani-Rahbar, Marian L. Neuhouser, Judd L. Walson

**Affiliations:** 1 Department of Epidemiology, University of Washington, Seattle, WA, United States of America; 2 Department of Environmental and Occupational Health Sciences, University of Washington, Seattle, WA, United States of America; 3 Centre for Global Health Research, Kenya Medical Research Institute, Kisumu, Kenya; 4 Paul G. Allen School for Global Animal Health, Washington State University, Pullman, WA, United States of America; 5 Global Disease Detection, Centers for Disease Control and Prevention, Nairobi, Kenya; 6 Department of Biostatistics, University of Washington, Seattle, WA, United States of America; 7 Division of Public Health Sciences, Fred Hutchinson Cancer Research Center, Seattle, WA, United States of America; 8 Department of Global Health, University of Washington, Seattle, WA, United States of America; TNO, NETHERLANDS

## Abstract

Livestock ownership has the potential to improve child nutrition through various mechanisms, although direct evaluations of household livestock and child stunting status are uncommon. We conducted an analysis of Demographic and Health Survey (DHS) datasets from Ethiopia (2011), Kenya (2008–2009), and Uganda (2010) among rural children under 5 years of age to compare stunting status across levels of livestock ownership. We classified livestock ownership by summing reported household numbers of goats, sheep, cattle and chickens, as well as calculating a weighted score to combine multiple species. The primary association was assessed separately by country using a log-binomial model adjusted for wealth and region, which was then stratified by child diarrheal illness, animal-source foods intake, sub-region, and wealth index. This analysis included n = 8079 children from Ethiopia, n = 3903 children from Kenya, and n = 1645 from Uganda. A ten-fold increase in household livestock ownership had significant association with lower stunting prevalence in Ethiopia (Prevalence Ratio [PR] 0.95, 95% CI 0.92–0.98) and Uganda (PR 0.87, 95% CI 0.79–0.97), but not Kenya (PR 1.01, 95% CI 0.96–1.07). The weighted livestock score was only marginally associated with stunting status. The findings varied slightly by region, but not by wealth, diarrheal disease, or animal-source food intake. This analysis suggested a slightly beneficial effect of household livestock ownership on child stunting prevalence. The small effect size observed may be related to limitations of the DHS dataset or the potentially complicated relationship between malnutrition and livestock ownership, including livestock health and productivity.

## Introduction

More than a quarter of the world’s children suffer from chronic malnutrition, resulting in linear growth failure (stunting), cognitive delay and increased risk of morbidity and mortality [1]. These consequences have profound implications for population health and economic improvement, as adults who were stunted as children have been shown to receive less education and achieve lower earnings [2, 3]. Rural areas in many resource-limited settings shoulder an unequal burden of malnutrition; rural children have nearly fifty percent higher stunting prevalence than children in urban areas [4]. In order to address stunting where the need is greatest, appropriate interventions must be developed which will be adopted by the families based on individual and community priorities [5].

Rural children commonly live in close proximity to livestock. These livestock may be an important determinant of child nutritional status, and promoting livestock production is a common development strategy. However, the overall influence of livestock ownership on child nutrition is not well understood as very few studies have examined the direct effect. A recent cross-sectional study in Kenya showed a small benefit of overall livestock ownership on child weight [6]. This effect could be mediated through livestock serving as direct sources of protein through meat, milk, and eggs or indirectly by increasing household income for food expenditure. An analysis of survey data in Uganda showed higher consumption of animal source foods in households with more livestock ownership, although this did not translate strongly into improved child growth outcomes [7]. However, randomized trials have also demonstrated that animal-source foods can improve weight gain and muscle development in children [8, 9].

Livestock ownership may also increase exposure to environmental contamination with fecal material and zoonotic pathogens [10, 11]. These exposures can lead to growth stunting by increasing overall metabolic demand, decreasing appetite, and by causing inflammatory patterns that reduce enteric absorption of nutrients [12, 13]. Fecal pathogens are closely linked to diarrheal illness, which has been directly related to stunting in children in low resource settings [14]. Finally, environmental enteric dysfunction, as a result of exposure to contaminated environment, appears to be strongly related to linear growth failure [13, 14]. Understanding the effect of livestock on child growth may provide opportunities for interventions in animals to improve healthy development of young children.

The Demographic and Health Surveys (DHS) provide publicly available datasets designed to monitor health status in over 90 countries at 5-year intervals. These rich datasets include both child growth and livestock ownership information. Anthropometric measures are recorded from children under 5 years of age and are often used to assess national burden of malnutrition. Several studies have evaluated the cross-sectional risk factors for child stunting using DHS data and have generally reported that greater wealth, education, and sanitation are associated with lower stunting prevalence [15–17]. The DHS also collects data on livestock ownership at the household level, although this measure is not routinely evaluated in association with child stunting.

Using existing DHS data from Ethiopia, Kenya, and Uganda, we sought to determine whether these publicly available cross-sectional data demonstrate a relationship between livestock ownership and child stunting prevalence, and whether these data can identify subgroups of households that could benefit from a targeted animal-human health intervention.

## Materials and Methods

### Datasets

This study was conducted as a regional analysis of the most recent DHS datasets from three East African countries, including Ethiopia (2011), Kenya (2008–2009), and Uganda (2010). Overall data collection methods for each DHS involved two-stage cluster designs and are described elsewhere [18]. In Ethiopia, a representative sample of 17,817 households was selected for the 2011 DHS [19]. For Kenya, a nationally representative sample of 8,444 women and 3,465 men was selected from 400 clusters throughout Kenya, which provided representative estimates for eight subdivisions [20]. The Ugandan survey included a representative sample of 10,086 households [21]. Data were restricted to rural households as the relationship between animals, wealth, and nutrition may be very different in urban households. Children sampled in these households were between 0 and 59 months of age.

### Variable definitions

Consistent with WHO practices, we categorized children as stunted if they had a height-for-age z score of less than −2 standard deviations below the WHO 2006 reference mean [22]. Children were only included in the analysis if they had a value for both height and age.

Livestock ownership numbers, including number of cattle, chickens, sheep, and goats, are included in the DHS as an asset available for use in the principal components analysis of the wealth index. Horses and donkeys are also enumerated in the dataset, but animals which are owned mainly for the purpose of load-bearing were not included in this analysis. Although camels can be an important livestock species in many areas throughout Ethiopia and Kenya, they were only available in the Ethiopia dataset and so were not analyzed here. The use of livestock as an exposure for human health outcomes is uncommon, so we applied three exploratory approaches for exposure measurement. First we created a total sum variable which gave each livestock species equal weight. This variable was highly positively skewed with most families owning small numbers of livestock, so a natural log was calculated. Second, we used counts of livestock as separate species. Third, a weighted measure of livestock was calculated using a Tropical Livestock Unit (TLU) scores. The TLU is a metric developed by the Food and Agriculture Organization (FAO), which allows for the combination of multiple species of livestock into a weighted measure representing total body weight and potentially market value. A single animal weighing 250kg represents a single TLU, providing weighting factors of 0.7 for cattle, 0.1 for sheep, 0.1 for goats, and 0.01 for chickens [23]. As with animal ownership counts, this measure was also positively skewed, and we created five categories of TLU ownership to be roughly reflective of potential differing household livestock composition.

To identify if animal ownership was associated with a stronger or weaker effect on stunting in certain subgroups of children, we stratified the association between livestock ownership and child stunting by diarrheal disease, region, wealth index (not including animals), and animal source food (ASF) intake. These variables were chosen *a priori* as categories by which the relationship between livestock and child stunting might be modified (e.g., if the effect of animal ownership is mediated through ASF, then those children who are fed ASF and have livestock may have a stronger positive effect). Recent diarrheal disease was defined as a caregiver’s positive response to the question “Has (NAME) had diarrhea in the past two weeks?” where 3 loose stools within a single day were considered diarrhea. Region was defined by the data collection team as the location where the household was sampled. Regions in Kenya have recently been redefined to reflect county-level governments, but due to the time of this survey, original province designations were retained. Children were considered to have consumed ASF if the child’s caregiver answered “yes” to whether the child ate eggs, meat, organ-meat, or dairy products in a 24-hour food recall. Although child consumption of individual ASF components and general dietary diversity are both important feeding indicators for child growth, these measures were not available in all datasets and therefore were not included in the analysis.

To create a wealth index which did not include livestock, we conducted a Principal Components Analysis (PCA) using indicators for use of surface water, the time it takes to obtain water, roof type, floor type, number of people per room, household electricity, television ownership, refrigerator ownership, bicycle/motorcycle/car ownership, telephone ownership, mobile phone ownership, use of shared toilet, and amount of land owned [24]. We used the first component score to create a quintile measure of wealth. We also included a binary indicator for maternal education as a further adjustment for confounding. This indicator was defined as yes if the child’s mother completed any education.

### Statistics

All analyses were conducted using Stata/SE 11 (StataCorp, LP). We described livestock and child health indicators by country using means and proportions. These were weighted by sample weights provided in the DHS dataset to account for sampling scheme which allowed for accurate national-level estimates. For proper weighting, the datasets were maintained separately by country.

To evaluate the association between household animal ownership and child stunting, we first visualized child stunting proportions across increasing categories of TLU within each country. We then formally assessed the relationship using General Estimating Equations (GEE). For this analysis, we included all children under age 5 in each dataset, which included some children living in the same household who were therefore exposed to the same levels of livestock ownership. To account for the correlation of stunting outcomes of children living in the same household, we used an exchangeable correlation structure. The GEE used a log-link and a binomial family to provide comparative prevalence ratios. This model did not use survey weights, in accordance with DHS data user recommendations [18]. After evaluating the univariable association, the association between livestock ownership and stunting was adjusted for wealth index and region. The model was further stratified by ASF, diarrheal disease, region, and wealth index. Hypotheses were tested using Wald statistics.

This analysis resulted in extensive multiple comparisons within the three datasets. Due to these multiple comparisons, we chose to adjust for False Discovery Rate (FDR) using the Benjamini-Hochberg method, as a less-conservative method compared to the Bonferroni method [25]. All of the subgroup analyses in each country dataset were considered part of a total of 97 tests. We used this total number of tests to correct the statistical significance cut-off in order to control the percentage of significant results that were false positives. Using this method, the highest p-value was compared to a cut-off of 0.05, but the lowest p-value was compared to a cut-off of 0.0005.

This study used publicly-available de-identified data, for which IRB review was not necessary from the University of Washington, nor from the coauthors’ institutions.

## Results

Among 8720 children from Ethiopia, 8079 had height measurements among 5528 households. In Kenya, 3903 children (of 4203 total children) in 2602 households had height measurements, and in Uganda 1645 in 1025 households (of 1740 total children) had height measurements. Stunting prevalence was high in each country at 29.1% in Kenya, 29.4% in Uganda, and 39.7% in Ethiopia (Table 1). Livestock ownership was variable by country, as well as by region within the country. Overall, Ethiopia had the highest number of animals per household. Within countries, the regions in northern Ethiopia, northern Uganda, and northeastern Kenya had higher average herd sizes than in the southern regions. The highest mean number of animals per household in Ethiopia and Kenya was goats, while in Uganda it was chickens. However, the most commonly owned animal in both Kenya and Uganda was chickens (63.2% and 60.3% of households owned chickens). The most commonly owned animals in Ethiopia were cattle, which were owned by 71.8% of the households.

**Table 1:** Household and child characteristics from Ethiopia 2011, Kenya 2008–2009 and Uganda 2010 Demographic and Health Surveys (DHS).

Characteristic	Ethiopia (N = 8720)	Kenya (N = 4203)	Uganda (N = 1740)
Household indicators			
Tropical livestock unit, mean(SD^a^)	2.34 (0.10)	1.55 (0.21)	0.90 (0.09)
Chicken count, mean (SD)^b^	3.18 (0.13)	5.91 (0.36)	5.25 (0.29)
Cow count, mean (SD) ^b^	3.41 (0.15)	1.53 (0.28)	1.50 (0.18)
Sheep count, mean (SD) ^b^	1.92 (0.19)	3.78 (0.63)	0.56 (0.11)
Goat count, mean (SD) ^b^	4.27 (0.42)	6.10 (0.85)	2.22 (0.13)
Wealth score, mean(SD)^c^	2.68 (0.04)	2.64 (0.07)	2.67 (0.06)
Child Indicators			
Height for age, mean Z-score (SD)	-1.53 (1.67)	-1.19(1.57)	-1.30 (1.46)
Stunting^d^, %(SD)	39.7% (0.01)	29.2% (0.01)	29.4% (0.02)
Recent diarrheal illness %(SD)	14.4% (0.01)	15.0% (0.01)	22.3% (0.01)

^a^ Standard deviation (SD)

^b^Among households which own any animals

^c^ No animals are included in the wealth score

^d^ Defined as height-for-age z-score lower than 2 standard deviations below the reference mean

**Fig 1:**
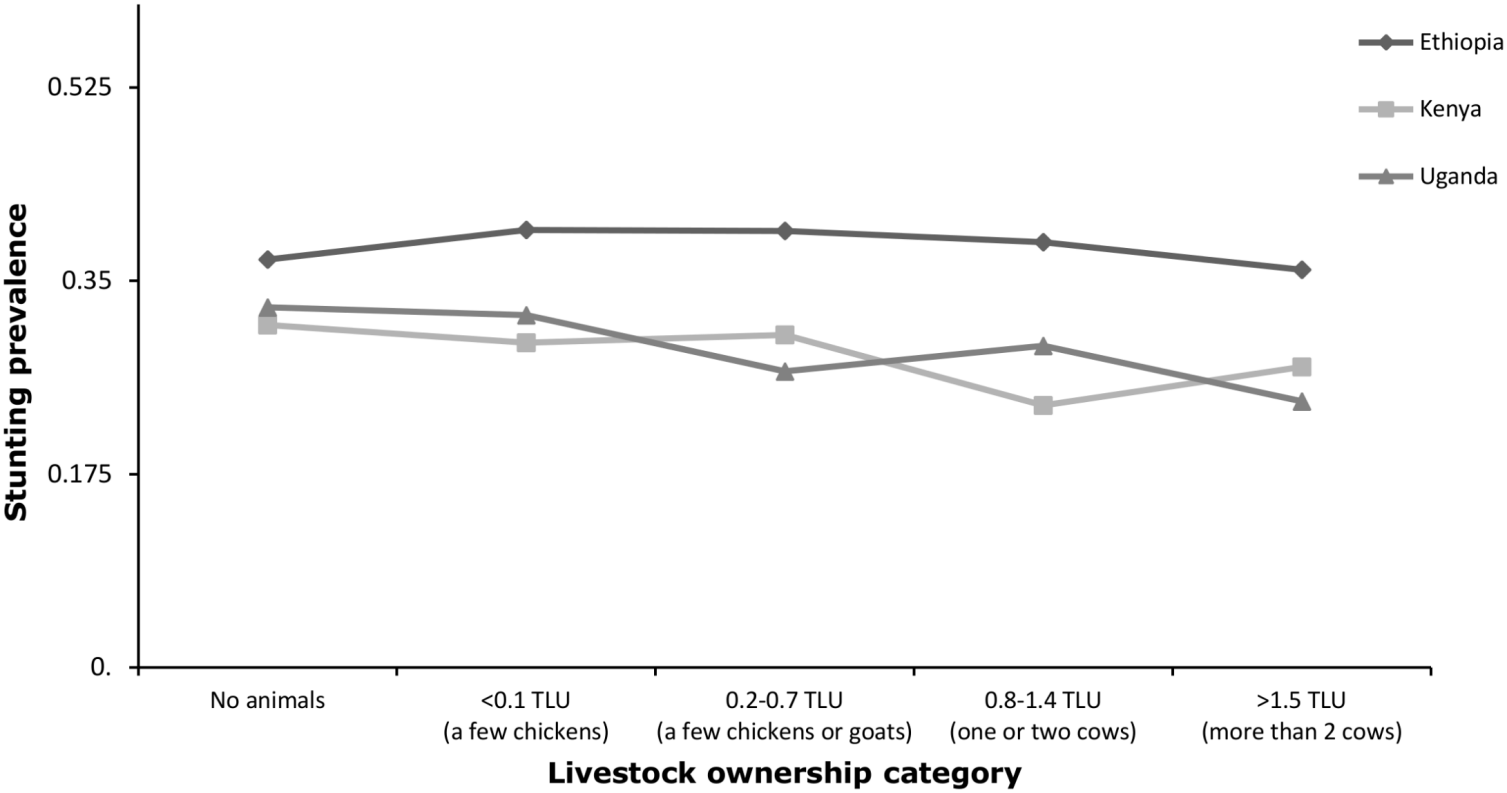
Unadjusted stunting prevalence by livestock ownership category in Ethiopia, Kenya, and Uganda.

Higher numbers of livestock and of Tropical Livestock Units (TLUs) were marginally associated with lower stunting prevalence (Table 2), although many of the associations were not statistically significant. Log livestock count was associated with decreased stunting prevalence in Ethiopia (adjusted PR 0.95, 95% CI 0.92–0.98) and Uganda (adjusted PR 0.87, 95% CI 0.79–0.96), but not in Kenya (PR 1.02, 95% CI 0.97–1.07). Higher TLU households trended towards lower stunting prevalence, but the association was not significant at a cut-off value of α = 0.05. Stunting prevalence decreased slightly by TLU livestock category, but the trend was not significant (Fig 1).

**Table 2:** Log-binomial models for the relationship between household livestock ownership and stunting prevalence in Ethiopia, Kenya, and Uganda.

Model	Prevalence Ratio Estimate (95% Confidence Interval)		
	Ethiopia	Kenya	Uganda
Log total livestock, unadjusted	0.98 (0.95–1.01)	1.02 (0.97–1.08)	0.87 (0.79–0.96)
Log total livestock, adjusted*	0.95 (0.92–0.98)	1.01 (0.96–1.07)	0.87 (0.79–0.97)
TLU**, unadjusted	0.99 (0.98–1.00)	1.00 (0.99–1.01)	0.92 (0.87–0.99)
TLU, adjusted*	0.99 (0.98–1.00)	1.00 (0.98–1.01)	0.94 (0.88–1.00)
TLU category, adjusted*			
No animals (ref)	-	-	-
<0.1 TLU (a few chickens)	1.10 (0.94–1.28)	0.97 (0.83–1.13)	0.99 (0.78–1.24)
0.2–0.7 TLU (chickens or goats)	1.03 (0.92–1.15)	0.96 (0.84–1.10)	0.81 (0.66–0.98)
0.8–1.4 TLU (one or two cows)	0.98 (0.87–1.10)	0.80 (0.66–0.98)	0.91 (0.68–1.22)
>1.5 TLU (more than 2 cows)	0.89 (0.80–1.00)	0.92 (0.78–1.09)	0.80 (0.61–1.05)

*Adjusted for wealth score, education, and region

**Tropical Livestock Unit (TLU): weighted livestock score combining chickens, cows, sheep, and goats

After adjusting for wealth, recent diarrheal illness in children was associated with an increased prevalence of stunting in Ethiopia (adjusted PR 1.10, 95% CI 1.02–1.08) and Kenya (adjusted PR 1.13, 95% 1.00–1.27), but the association was not significant in Uganda (PR 1.14, 95% CI 0.96–1.35). Similarly, increased asset-based wealth score was associated with decreased stunting prevalence in Ethiopia (PR 0.94, 95% CI 0.92–0.97) and Kenya (PR 0.87, 95% CI 0.84–0.91), but not in Uganda (PR 0.97, 95% CI 0.91–1.03). The measure for 24-hour recall of ASF intake was not found to be associated with stunting in any country.

The association between TLUs and stunting prevalence did not vary by wealth index or diarrheal illness (Table 3). In Ethiopia, the association differed across region; in Dire Dawa an increase of one TLU was associated with 22% less stunting (Table 4). The child’s 24-hour ASF intake did not modify the effect between the livestock and stunting prevalence in any country (data not shown). In each country, we also evaluated whether ownership of cattle, goats, sheep, or chickens as individual species were related to child stunting. Holding constant wealth, region and other animal ownership, none of these species was independently related to stunting status.

**Table 3:** Log-binomial models for the relationship between household livestock ownership and stunting prevalence, as stratified by wealth and two-week diarrheal disease history.

		Prevalence Ratio Estimate (95% Confidence Interval)		
Model	Level	Ethiopia	Kenya	Uganda
TLU* stratified by wealth index	Poorest	0.99 (0.97–1.01)	1.01 (0.99–1.02)	0.88 (0.72–1.07)
	Poorer	0.99 (0.97–1.01)	1.01 (0.98–1.03)	0.75 (0.61–0.94)
	Moderate wealth	0.95 (0.92–0.99)	0.89 (0.80–1.00)	0.98 (0.84–1.13)
	Wealthier	0.99 (0.96–1.01)	0.85 (0.75–0.97)	0.99 (0.90–1.10)
	Wealthiest	1.03 (0.96–1.08)	1.04 (0.95–1.15)	0.97 (0.87–1.08)
	Interaction	p = 0.576	p = 0.131	p = 0.022
TLU stratified by diarrheal illness	(Yes)	0.98 (0.97–1.00)	1.00 (0.99–1.02)	0.93 (0.86–1.00)
	(No)	1.01 (0.98–1.03)	0.99 (0.96–1.03)	0.97 (0.87–1.07)
	Interaction	p = 0.065	p = 0.961	p = 0.607

*Tropical Livestock Unit (TLU): weighted livestock score combining chickens, cows, sheep, and goats

**Table 4:** Log-binomial models for the relationship between household livestock ownership and stunting prevalence, as stratified by region.

Ethiopia		Kenya		Uganda	
Region	TLU^a^ PR^b^ (95% CI^c^)	Region	TLU PR (95% CI)	Region	TLU PR (95% CI)
Tigray	0.99 (0.96–1.03)	Central	0.84 (0.64–1.09)	Central 1	0.91 (0.66–1.26)
Afar	1.00 (0.99–1.01)	Coast	0.98 (0.85–1.14)	Central 2	0.90 (0.71–1.13)
Amhara	0.97 (0.93–1.01)	Eastern	1.01 (0.99–1.03)	East Central	1.11 (0.94–1.31)
Oromiya	0.93 (0.89–0.98)	Nyanza	0.96 (0.81–1.14)	Eastern	0.92 (0.77–1.11)
Somali	0.99 (0.95–1.04)	Rift Valley	0.99 (0.96–1.02)	North	0.78 (0.54–1.13)
Benishangul-gumuz	0.96 (0.91–1.02)	Western	0.78 (0.50–1.20)	Karamoja	0.95 (0.80–1.10)
SNNPR	0.92 (0.88–0.97)	Northeastern	0.99 (0.97–1.01)	West-Nile	0.92 (0.79–1.08)
Gambela	1.01 (0.99–1.04)	Interaction	p = 0.355	Western	1.01 (0.91–1.12)
Harari	1.06 (0.90–1.24)			Southwest	0.63 (0.39–1.00)
Dire Dawa	0.78 (0.67–0.91)			Interaction	p = 0.389
Interaction	p = 0.0004				

^a^Tropical Livestock Unit (TLU): weighted livestock score combining chickens, cows, sheep, and goats

^b^Prevalence Ratio (PR)

^c^Confidence Interval (CI)

Although several of these tests suggested small but statistically significant impact of animal ownership on stunting using a universal p-value = 0.05, when the Benjamini-Hochberg method to adjust for multiple comparisons was applied, only three regional associations in Ethiopia remained statistically significant (Oromiya, SNNPR, and Dire Dawa). None of the overall associations between livestock and stunting were strong enough to remain significant after adjusting for multiple comparisons.

## Discussion

This analysis provides an initial approach towards understanding child stunting and livestock ownership at the household level in rural Eastern Africa. Livestock are nearly ubiquitous in these households and have many potential relationships with child growth. In most of the analyses presented here, the relationship between livestock ownership and child stunting demonstrated a trend towards a protective association. Only a few other studies have examined the overall association of livestock ownership and stunting, and prior reports have been consistent with our findings of a small effect sizes [6, 26]. These results suggest that there may be room to improve the extent to which livestock provide benefit to child nutrition. Additional research is necessary to understand the specific populations for whom and methods by which livestock can benefit child growth.

Healthy child growth and development is contingent on a delicate balance between optimal diet, sanitation and overall health. As such, stunting in children could be the result of protein or calorie insufficiency, micronutrient deficiency, repeated infection, environmental enteropathy, or intra-uterine growth restriction [5, 13, 14, 27]. Household livestock may be related to many of these causes. Livestock can have positive impact on macro and micronutrient deficiency through animal-source food provision. However, if families do not utilize animal-source foods, the livestock cannot provide this direct benefit. In this analysis, 24 hour recall of animal-source foods was not related to stunting status or livestock ownership, which may partially explain the lack of significant benefit. However, the animal-source foods measure itself was only a cross-sectional 24 hour yes/no recall of intake, which may be an unstable estimate of true consumption.

Livestock may also be related to infectious disease among children. Livestock-associated wealth may allow households greater ability to access services such as improved water and sanitation, or health care. As a result, ownership of healthy livestock may improve the child’s environment and decrease the impact of infectious diseases that would otherwise lead to stunting. However, it is plausible that sick or asymptomatically infected livestock could also transmit zoonoses and add to contamination. This pathway has not been well-evaluated and further research is needed. Interventions addressing the complex nature of livestock, sanitation and veterinary care practices could be optimized to improve child nutrition and growth in these settings.

The small effect size shown here may suggest a truly negligible relationship between livestock and child stunting, as there are some causes of stunting which would not be related to childhood environmental conditions. For example, children who were born small-for-gestational age comprise up to 20% of children who are stunted between ages 1 and 5 years [27] and these children may not be impacted by livestock ownership during childhood. In addition, previous studies have suggested that a very minimal proportion of stunting can be reversed with optimal nutritional intervention, suggesting that interventions such as livestock provision may be unable to dramatically alter the progression of linear growth failure in these children [5]. In these instances, stunting status may not show the full benefit of livestock ownership. Additional outcomes such as cognitive development and educational attainment may be importantly linked to livestock ownership, but we are unable to evaluate these outcomes with the DHS dataset.

Finally, from a statistical standpoint, these DHS datasets provide national-level data from multiple years. The associations reported here are averaged over many groups of people, each with different relationships with their animals. Fortunately, large datasets such as these also provide the opportunity to identify subgroups in which the effect size might be larger or smaller. In this analysis, effect modification, other than by sub-region, was not strongly apparent. Additionally, the DHS data were not collected to analyze livestock ownership patterns. The DHS collects animal ownership data for the purpose of creating a wealth index, and thus the exposure measures are not sufficiently comprehensive enough to distinguish nuances of the patterns of livestock ownership and child growth. There is no estimate of household ASF production levels, and the feeding measures are restricted. However, because it is a health survey, the outcomes in the DHS are at the child level, which allows for an analysis of child nutrition that is not possible using several extant livestock datasets which lack human data. This analysis is also limited in that it is cross-sectional. A longitudinal survey would allow for a temporal understanding of the influence of livestock on child growth, such as whether higher livestock ownership is important during critical growth windows, or whether livestock influence growth velocity.

Livestock production improvement programs are common in rural areas of developing countries as a means to promote income generation and improve nutritional status. Further, many large international charities provide assistance to rural families in the form of livestock gifts. Although this study did not formally evaluate this practice, it suggests that animal ownership alone may have only a small influence on the prevalence of stunting among young children. Longitudinal trials of livestock donation on nutritional outcomes would help to inform these interventions to ensure the maximum benefit. Potential ways to enhance livestock production interventions could include provision of education on ASF feeding practices, ensuring veterinary care for animals, and promoting livestock sanitation. Although national-level data can give some insight into the overall influence of livestock ownership on child stunting status, further specific studies with greater resolution on household livestock ownership and production are necessary to dissect these relationships.
